# Distinct projection patterns of different classes of layer 2 principal neurons in the olfactory cortex

**DOI:** 10.1038/s41598-017-08331-0

**Published:** 2017-08-15

**Authors:** Camille Mazo, Julien Grimaud, Yasuyuki Shima, Venkatesh N. Murthy, C. Geoffrey Lau

**Affiliations:** 1 Department of Molecular and Cellular Biology and Center for Brain Science, Harvard University, Cambridge, MA USA; 2 Ecole Normale Supérieure de Cachan, Université Paris-Saclay, F-94235 Cachan, France; 3 Department of Biology, Brandeis University, Waltham, MA 02454 USA; 4 Department of Biomedical Sciences and Centre for Biosystems, Neuroscience and Nanotechnology, City University of Hong Kong, 83 Tat Chee Avenue, Kowloon Tong, Hong Kong

## Abstract

The broadly-distributed, non-topographic projections to and from the olfactory cortex may suggest a flat, non-hierarchical organization in odor information processing. Layer 2 principal neurons in the anterior piriform cortex (APC) can be divided into 2 subtypes: semilunar (SL) and superficial pyramidal (SP) cells. Although it is known that SL and SP cells receive differential inputs from the olfactory bulb (OB), little is known about their projections to other olfactory regions. Here, we examined axonal projections of SL and SP cells using a combination of mouse genetics and retrograde labeling. Retrograde tracing from the OB or posterior piriform cortex (PPC) showed that the APC projects to these brain regions mainly through layer 2b cells, and dual-labeling revealed many cells extending collaterals to both target regions. Furthermore, a transgenic mouse line specifically labeling SL cells showed that they send profuse axonal projections to olfactory cortical areas, but not to the OB. These findings support a model in which information flow from SL to SP cells and back to the OB is mediated by a hierarchical feedback circuit, whereas both SL and SP cells broadcast information to higher olfactory areas in a parallel manner.

## Introduction

Sensory perception emerges from the confluence of bottom-up and top-down inputs. In olfaction, feedback projections innervate the first brain relay for information processing: the olfactory bulb (OB). The OB receives information from olfactory receptor neurons, each bearing a single odorant receptor but expressing ~1,000 odorant receptors altogether in mice^1, 2^. All sensory neurons expressing the same receptor converge to ~2 glomeruli within each OB^3^, where they synapse onto apical dendrites of OB principal cells (mitral and tufted cells) as well as glomerular layer interneurons, thereby forming a map of receptor identity. A given OB principal cell sends its apical dendrite to a single glomerulus, while the populations of mitral and tufted cells multiplex odor information to a variety of higher brain regions, including the anterior piriform cortex (APC)^4–6^. The APC is the largest region of primary olfactory cortex. It is thought to be involved in odor identity encoding, and to serve as a location for learning-induced changes in olfaction^7, 8^. Single piriform neurons receive convergent inputs from multiple glomeruli. At the population level, odor information in the APC is sparse and distributed, and lacks evident topographic organization^5, 6, 9–13^. Odor information encoded by assemblies of APC cells is then transmitted to a variety of olfactory regions such as the anterior olfactory nucleus (AON), posterior piriform cortex (PPC), cortical amygdala (CoA), and lateral entorhinal cortex (LEnt). These olfactory cortical areas also project to higher, non-sensory brain regions such as the orbitofrontal cortex (OFC). However, little is known about the organization of APC projection channels.

The APC is a paleocortex composed of three layers. From superficial to deep: layer 1 is the input layer, layer 2 contains densely packed principal cells, and layer 3 comprises a combination of principal cells and GABAergic neurons. Deep to layer 3 is the endopiriform cortex (EndoP), mainly populated with multipolar neurons^14^. Furthermore, layer 2 can be divided into two sublayers, 2a being roughly the superficial half of layer 2, and 2b the deeper half. Afferent inputs from the OB make synapses mainly with the distal dendrites of layer 2 principal cells. However, the strength and connectivity of these synapses appear to be cell-type specific: the semilunar (SL) cells in L2a receive stronger inputs while the superficial pyramidal (SP) cells in L2b receive weaker sensory inputs^15, 16^. In addition to these synaptic properties, recent work demonstrated that SL and SP cells exhibit cell-type specific connectivity^17, 18^. SL cells make synapses onto layer 2b SP cells without forming recurrent synapses on to themselves, while SP cells are recurrently connected^17^. Therefore, layer 2 is populated with a mix of principal cells, namely SL and SP cells^16^, playing different roles in the synaptic processing of olfactory information^15^.

Input processing and recurrent connectivity is well described in the APC^4, 8, 15, 19^. However, it is unclear which neuron types contribute to the numerous projections out of the APC. Reconstruction studies of individual neurons suggest that APC principal cells project axons to the OB, AON, and to downstream olfactory regions such as the PPC, LEnt, and CoA^20, 21^. However, it is unclear how prevalent cells projecting both in feedforward and feedback directions are. Recent work^22, 23^ confirmed original findings from Haberly and Price^24^, showing that feedback fibers from the APC to the OB do not originate homogeneously from all layers but appear to come from layers 2b and layer 3. In the present work, we used Retrobeads, viral labeling, as well as mouse genetics to dissect the contribution of APC to upstream or downstream projections, with an emphasis on layer 2 principal neuron populations. We found that layer 2b is the main source of both feedback and feedforward projections, and that a sizeable fraction of neurons send collaterals to both regions. In addition, we found that genetically labeled SL cells projects widely to olfactory areas, but not back to the OB.

## Results

### The distribution of APC cells projecting back to the OB is biased toward layer 2b

**Figure 1:**
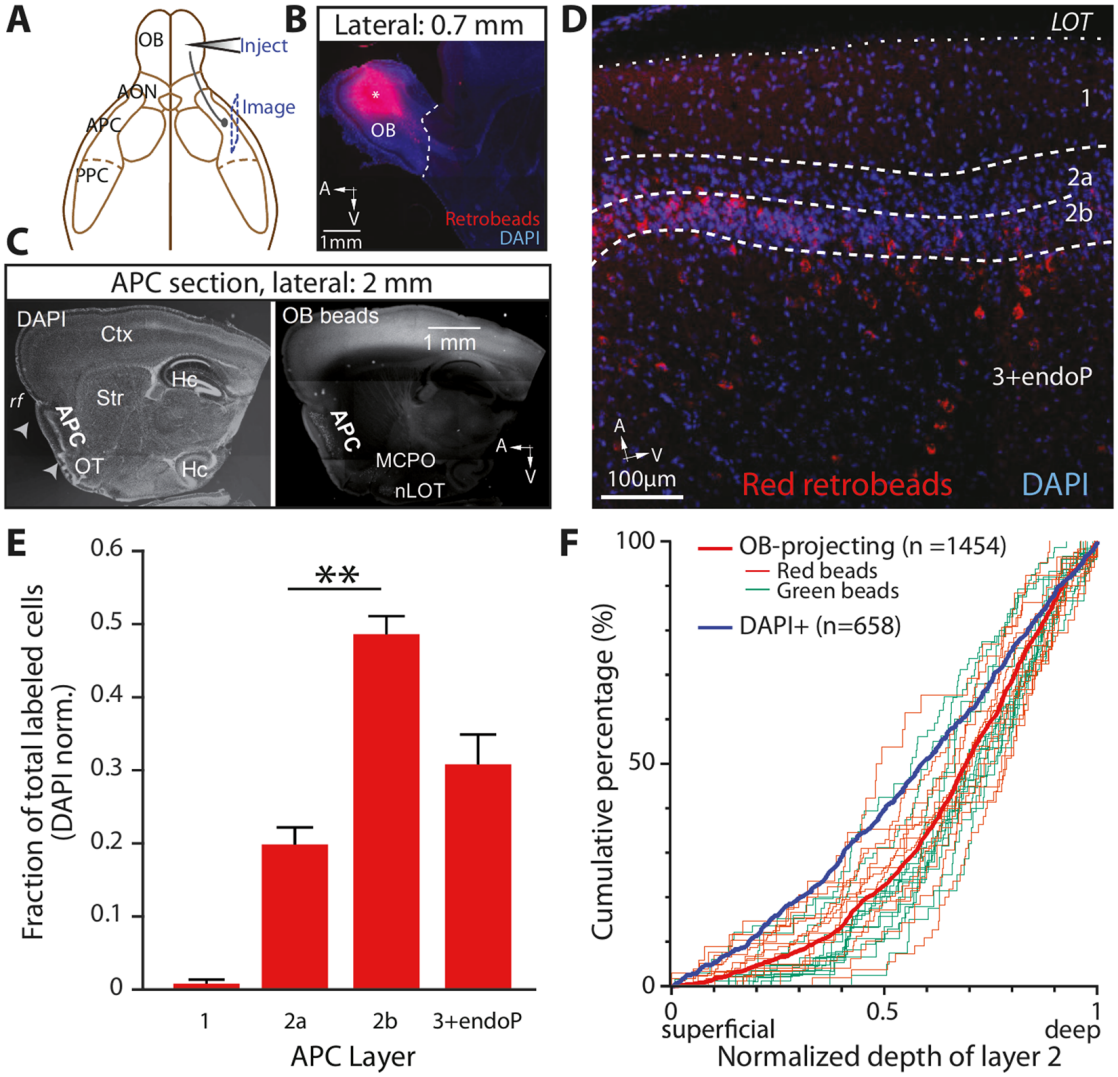
Layer 2b is the main source of feedback from the APC to the OB. (**A**) Schematic representation of injection of the tracer into the OB, and imaging from sagittal sections of the APC. (**B**) Injections were targeted to the granule cell layer of the OB. *, injection site. Red: retrograde tracer; blue: DAPI. (**C**) Representative sagittal section used for APC imaging. *Left*, DAPI labeling shows the main anatomical landmarks: dense cell layer 2 of the APC; rf: rhinal fissure; OT: olfactory tubercle; Hc: hippocampus; Ctx: neocortex and Str: striatum. *Right*, Retrogradely-labeled cells were found mainly in the APC in those sections. Some labeling was also observed in the MCPO: magnocellular preoptic nucleus and nLOT: nucleus of the lateral olfactory tract. (**D**) Higher magnification image showing retrogradely labeled cells across APC layers. Superficial limit of the layer 2 (border between layers 1 and 2a) is defined with a depth of 0 while a depth of 1 is the deep end of that layer (limit between layers 2b and 3). Red: retrograde tracer. Blue: DAPI. (**E**) Bar graph showing the relative fractions of retrogradely labeled neurons, normalized by the number of DAPI cells in each layer. OB-projecting neurons were heterogeneously distributed across APC layers (p < 0.0001, total cell count: 546 Retrobeads + , 4474 DAPI + cells, n = 13 sections, 11 mice, Friedman test). Within layer 2, cells were more densely found in layer 2b than in layer 2a (p = 0.005, n = 84 2a cells *vs*. 205 2b cells, Dunn’s multiple comparisons post-hoc test). **P < 0.01. (**F**) Cumulative distribution of the OB-projecting cells within layer 2. On the x-axis, 0 indicates the border between layers 1 and 2a, while 1 is the limit between layers 2b and 3 (see panel D). The light green and red curves show the results from the counting obtained in a single optical section with green and red Retrobeads, respectively. Distributions obtained from green and red Retrobeads were not significantly different (p = 0.47, Kolmogorov-Smirnov test, n = 856 cells, 14 sections, 6 mice for the green Retrobeads, n = 598 cells, 13 sections, 6 mice for the red Retrobeads). The distribution of all the bead-labeled cells (thick red trace) was shifted to deeper part of the layer 2 compared to the distribution of the DAPI + cells (thick blue trace; p < 0.0001, Kolmogorov-Smirnov test, n = 658 DAPI + cells).

To analyze the anatomical distribution of the somata of APC neurons projecting back to the OB, we injected retrograde tracers in the OB of mice (Fig. 1A,B) and examined the location of back-labeled somata in a series of sagittal sections of the APC **(**Fig. 1C **)**. We targeted our injections to the granule cell layer of the OB because it has been shown to be the largest recipient of feedback fibers originating from the APC^25–28^.

We injected either red or green fluorescent Retrobeads (50 nL) into the OB of C57Bl/6 J mice and imaged olfactory cortices 1 to 2 days later. Bead injections in the OB led to labeling profiles comparable to what has previously been described in the literature, notably with ipsilateral labeling of somata in the AON pars principalis, but not in the AON pars externa; and contralateral labeling in AON pars principalis and pars externa, and also labeling of the ipsilateral horizontal limb of the diagonal band of Broca^27–31^ (Supplementary Fig. S1). To examine whether particular neuron types are responsible for sending feedback projections to the OB, we analyzed the distribution of labeled neurons across APC layers (Fig. 1D). Following OB injections, retrogradely-labeled neurons were uniformly distributed along the medio-lateral (coronal sections) or antero-posterior (sagittal sections) axis of the ipsilateral APC. In contrast, across the different APC layers, we found a heterogeneous distribution of labeled cells, even when corrected for variation in cell densities across layers (p < 0.0001, Friedman test; Fig. 1E. See figure legends and Supplementary Table online for the detailed numbers). Since layer 2 can be subdivided into layer 2a (superficial) and 2b (deep) with different neuron types, we compared the relative density of labeled cells in these sublayers. The distribution of OB-projecting neurons was significantly biased toward layer 2b (19.8 ± 2.4% for layer 2a versus 48.6 ± 2.5% for layer 2b, p = 0.005, Dunn’s multiple comparisons post-hoc test; Fig. 1E), similar to results reported by Diodato and colleagues^22^, who did not normalize data to account for the variation in cell densities across layers. Interestingly, after normalization for sublayer cell densities, a substantial fraction of cells (33.7 ± 2.1%) was found in a continuum encompassing layer 3 and the endopiriform (endoP; Fig. 1E). Next, we further examined the distribution of retrogradely-labeled neurons as a function of the depth of layer 2 (0 being the superficial limit and 1 being the deep limit). First, we confirmed that red and green bead labeling led to a similar distribution to the deepest part of layer 2, and therefore data were pooled (p = 0.47, Kolmogorov-Smirnov test; Fig. 1F). Next, we found that the distribution of retrogradely-labeled neurons was significantly shifted toward the deepest part of layer 2 compared to the distribution of layer 2 cells measured using DAPI staining (p < 0.0001, Kolmogorov-Smirnov test; Fig. 1F).

However, our observations could have been influenced by the biased uptake of the Retrobeads by certain neuron types. To examine this, we injected adeno-associated viruses (AAVs) into the OB of mice expressing Cre under the CaMKIIa promoter (CaMKIIa-Cre), which is expressed in most excitatory principal neurons in the cortex^32^. Indeed, AAVs have recently be shown to possess retrograde-labeling activity, especially when used with transgenic mice expressing Cre recombinase in specific neural populations^33^. Our survey of AAV serotypes showed that AAV capsid serotype 8 (AAV2/8-CAG-DIO-EYFP) worked the best to retrogradely label APC somata using this in CaMKIIa-Cre mice (Supplementary Fig. S2). Consistent with bead injections, virally-mediated retrograde labeling of APC neurons was heterogeneous across layers (p = 0.0002, Friedman test; Supplementary Fig. S3A). Within layer 2, significantly more cells were found in layer 2b than in layer 2a (23.4 ± 4.1% for layer 2a versus 51.4 ± 0.8% for layer 2b, p = 0.018, Dunn’s multiple comparisons post-hoc test; Supplementary Fig. S3A).

Since both Retrobeads and virus injections into the OB labeled mostly L2b cells within L2, we controlled for any biased uptake by these cells by examining recurrent projections within the APC. Toward this aim, we injected Retrobead or AAV into the APC and imaged within the APC. This resulted in same amount of labeling in L2a vs. L2b after normalization for DAPI cell density for both retrograde tracers (virus injection: p = 0.50, Supplementary Fig. S3B; Retrobeads injection: p = 0.88, Supplementary Fig. S4A, Wilcoxon ranksum matched-pairs tests), showing that the Retrobeads and viruses are equally likely uptaken by L2a and L2b cells.

Using both Retrobeads and viral injections to label OB-projecting cells of the APC in a retrograde manner, we showed that the main OB-projecting population is located in layer 2b of the ipsilateral APC.

### The distribution of APC neurons projecting feedforward axons to the PPC is biased toward layer 2b

**Figure 2:**
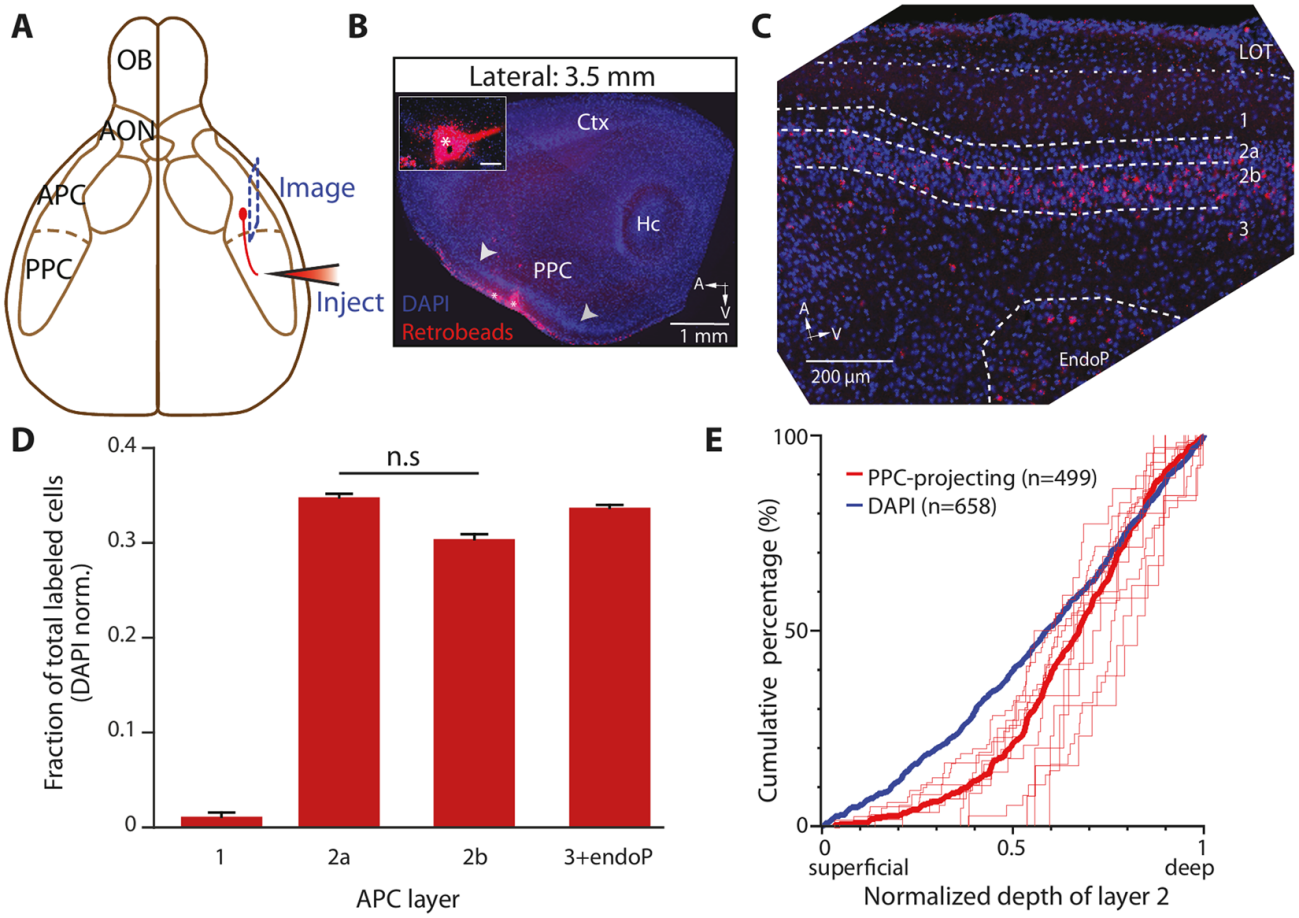
Projections from the APC to the PPC arise more homogeneously from both layer 2a and 2b. (**A**) Schematic representation of the injection of tracer into the PPC, and imaging from sagittal sections of APC. (**B**) Injections of tracer were targeted to the PPC. *, injection site. Arrowheads, PPC limits. Hc, hippocampus; Ctx, neocortex. *Inset*, zoom-in view of the injection site. Note the presence of LOT in the APC but not PPC. (**C**) Retrogradely labeled cells were found in APC sagittal sections. For B and C, red: tracer. Blue: DAPI. (**D**) Bar graph showing the relative fractions of retrogradely labeled neurons, normalized by the number of DAPI cells in each layer. PPC-projecting neurons were heterogeneously distributed across APC layers (p = 0.012, n = 581 Retrobeads + cells and 2529 DAPI + cells, 5 sections, 4 mice, Friedman test), with labeled cells mainly located in layer 2a, 2b and in layer 3 + EndoP. No statistical difference was found between layer 2a and 2b when fractions were corrected for variations in cell densities across layers (p = 0.46, n = 155 layer 2a cells and 191 layer 2b cells, Dunn’s multiple comparisons post-hoc test). n.s, not significant. (**E**) Within layer 2, projecting neuron distribution was skewed toward deeper part of layer 2 (p < 0.0001, n = 499 Retrobeads + neurons, n = 658 DAPI^+^ cells, 12 sections, 5 mice, Kolmogorov-Smirnov test). Thin red traces represent the counting for single sections, while the thick red trace shows the distribution of all the counted cells. The thick blue trace is the distribution of the DAPI + cells.

We then studied the distribution of the APC neurons projecting to the PPC, a major stream of feedforward information flow. Similar to OB injections, Retrobeads were injected into the PPC of mice and labeled somata were quantified in a series of sagittal APC sections (Fig. 2A–C). The distribution of PPC-projecting neurons across APC layers, corrected for the variation in cell densities, was also heterogeneous (p = 0.012, Friedman test; Fig. 2D). Interestingly, the non-normalized distribution was bimodal, with a peak in layer 2 (first peak at 74% relative to depth of L2; 57% of cells) and a smaller peak in the EndoP (second peak at 289% of L2; 26% of cells); substantially fewer cells were to be found in layer 3 compared to other layers (17% of cells; Supplementary Fig. S4B). These results corroborate the observations in an early work using horseradish peroxidase staining^24^. Within layer 2, the fraction of PPC-projecting cells was not significantly different between layer 2a and 2b after correction for variation in cell densities across layers (34.8 ± 2.1% in layer 2a *vs*. 30.4 ± 3.2% in layer 2b, p = 0.46, Dunn’s multiple comparisons post-hoc test; Fig. 2D). Yet the distribution of the PPC-projecting population was significantly skewed toward the deeper part of layer 2 compared to the distribution of the DAPI cells (p < 0.0001, Kolmogorov-Smirnov test; Fig. 2E). Thus, global, layer-wise analysis shows that PPC-projecting cells are equally dense in layer 2a and 2b, while finer, continuous analysis of their location revealed that PPC-projecting cell distribution is skewed toward deeper parts of layer 2 compared to the overall cell distribution. Finally, our experiments suggest that EndoP contains PPC-, but not OB-, projecting neurons, while layer 3 mostly contains OB-, but not PPC-, projecting neurons.

### OB- and PPC-projecting neurons are overlapping populations in layer 2 of the APC

**Figure 3:**
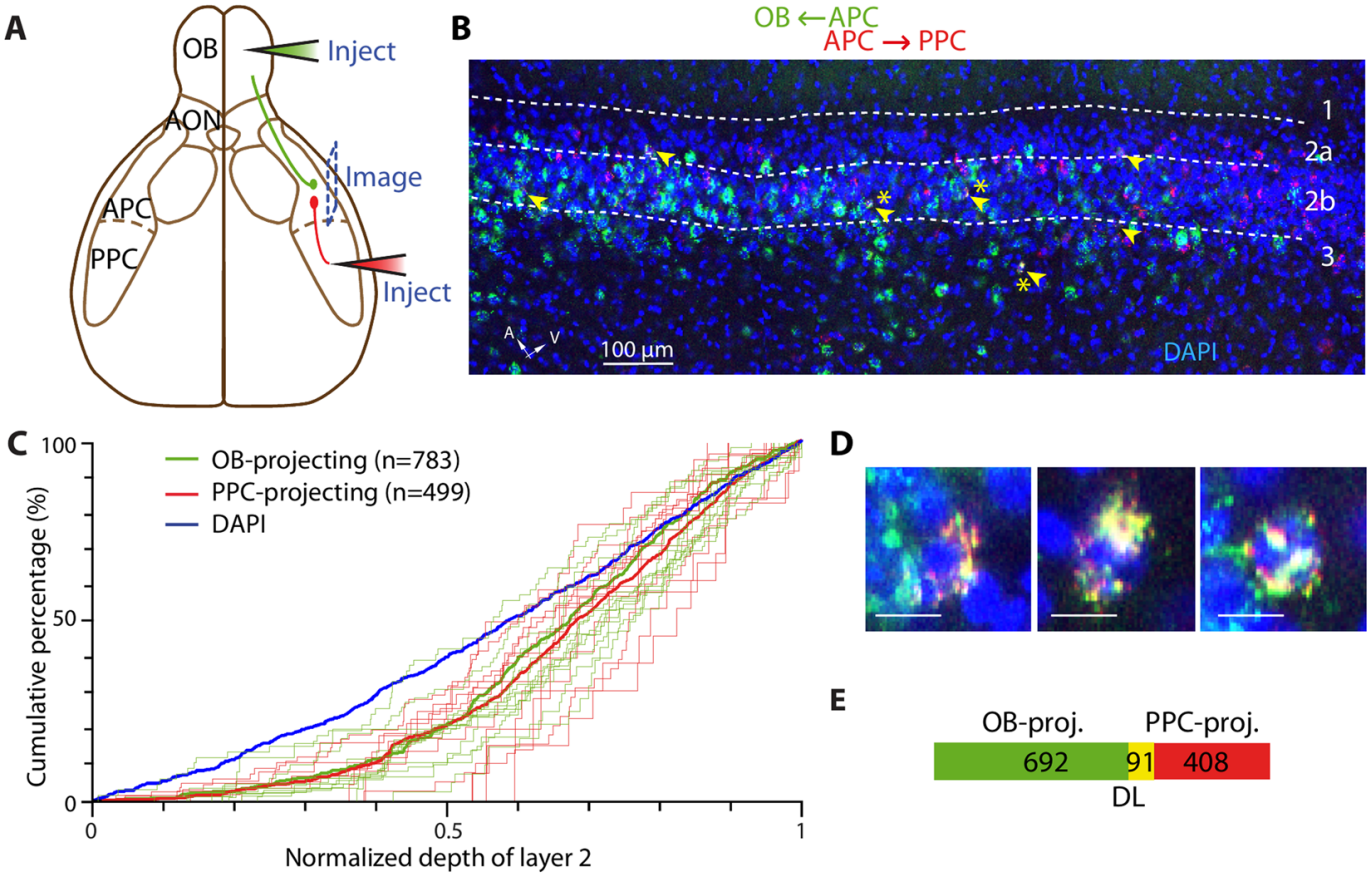
OB- and PPC-projecting cells are partially overlapping populations. (**A**) Schematic representation of the dual injection strategy. Green Retrobeads were injected into the OB while red Retrobeads were injected into the PPC. Images were taken from the APC. (**B**) Example APC section with OB- (green) and PPC-projecting (red) neuron population, spatially overlapping. Arrowhead: dual-labeled neurons. (**C**) OB- and PPC-projecting neurons share similar distributions within APC layer 2 (p = 0.052, n = 782 and n = 499 OB- and PPC-projecting neurons, respectively, 12 sections, 5 mice, Kolmogorov-Smirnov test). The thin green and red traces represent quantifications from single optical sections. The thick green and red traces are the distribution of all the OB- and PPC-projecting neurons respectively. (**D**) Blow-up of the starred arrows in B., showing 3 dual-labeled neurons in layer 2b. Scale bars: 10﻿ μm. (**E**) Venn diagram representing the counted number of dual-labeled cells (DL) in each population.

We observed that the OB- and PPC-projecting populations of the APC share dissimilar distribution patterns across the three layers and EndoP, but might share more similar patterns inside layer 2. We next asked whether the neurons from layer 2 projecting back to the OB and forward to the PPC belong to segregated or the same population of APC projecting cells, such that a single cell projects to both areas. Recent tracing work from Chen *et al*.^34^ showed that single APC neurons that project to two distinct areas of the OFC – namely the agranular insula and lateral OFC – were spatially segregated within the APC, suggesting a spatial organization of the APC based on its output channels. To address the spatial organization of OB- projecting cells relative to the PPC-projecting population and *vice versa*, we injected green Retrobeads in the OB and red Retrobeads in the PPC in the same mice (Fig. 3A). First, we did not find significant difference in the distribution patterns of OB-and PPC-retrogradely labeled neurons (p = 0.052, Kolmogorov-Smirnov test; Fig. 3B,C). Then, of the 783 retrogradely labeled neurons from the OB (green Retrobeads^+^), 91 of them (11.6%) were also projecting to the PPC (dual labeled). Similarly, 91 of the 499 cells projecting to the PPC were found to project to the OB as well (18.2%; Fig. 3B,D,E). The fact that we found non-zero percentages of dual-labeled cells shows that at least some of them project to both the PPC and OB.

Several factors may lead to an underestimation of the dual-labeled population. First, within the injection site, Retrobeads labeled only a fraction of neurons that actually project to the injected region. Moreover, dual dye injection might further result in low amount of dually-labeled cells, potentially due to competitive mechanism between dyes^35^. To estimate the dual-labeling efficiency, we successively injected identical amounts of green and red Retrobeads into the same site in the OB. Under these conditions, a large majority of labeled cells in the APC was double-labeled (90.5–92.7%; Supplementary Fig. S5A), similar to a previous study^35^. Thus, the co-labeling efficiency of green and red Retrobeads appears to be high and this factor only contributes weakly to the underestimation of dual-labeled population. Second, with Retrobeads, injections were spatially restricted to several hundred of micrometers in diameter (Figs 1A and 2A). Since we do not know the absolute number of APC neurons projecting to the OB or PPC, we cannot estimate the fraction of projecting cells we labeled using our bead injection protocol. Evidence for a certain degree of topography in OB-projection patterns^23, 36^ suggests that our restricted bead injection limits the number of possible retrogradely labeled neurons to those projecting to the precise injection locus. Notably, Matsutani^36^ described patchy, sparse axon terminals in the OB that originate from APC neurons. To appreciate the underestimation caused by restricted bead injection, we injected green and red Retrobeads into different sites within the OB (~500 µm apart) and observed co-labeling of only a third of the projecting neurons (21.1–34.3%; Supplementary Fig. S5B). Therefore, our results suggest that there was limited spread of the Retrobeads and our injection protocol largely underestimates the number of neurons projecting to the OB or the PPC. As a result, the actual dual-labeled population is likely to be much larger than what is estimated here. We conclude from these dual-labeling experiments that a sizeable fraction of layer 2 APC neurons project to both the OB and PPC.

### Output from layer 2a SL cells is widely distributed in olfactory areas but do not project to the OB

**Figure 4:**
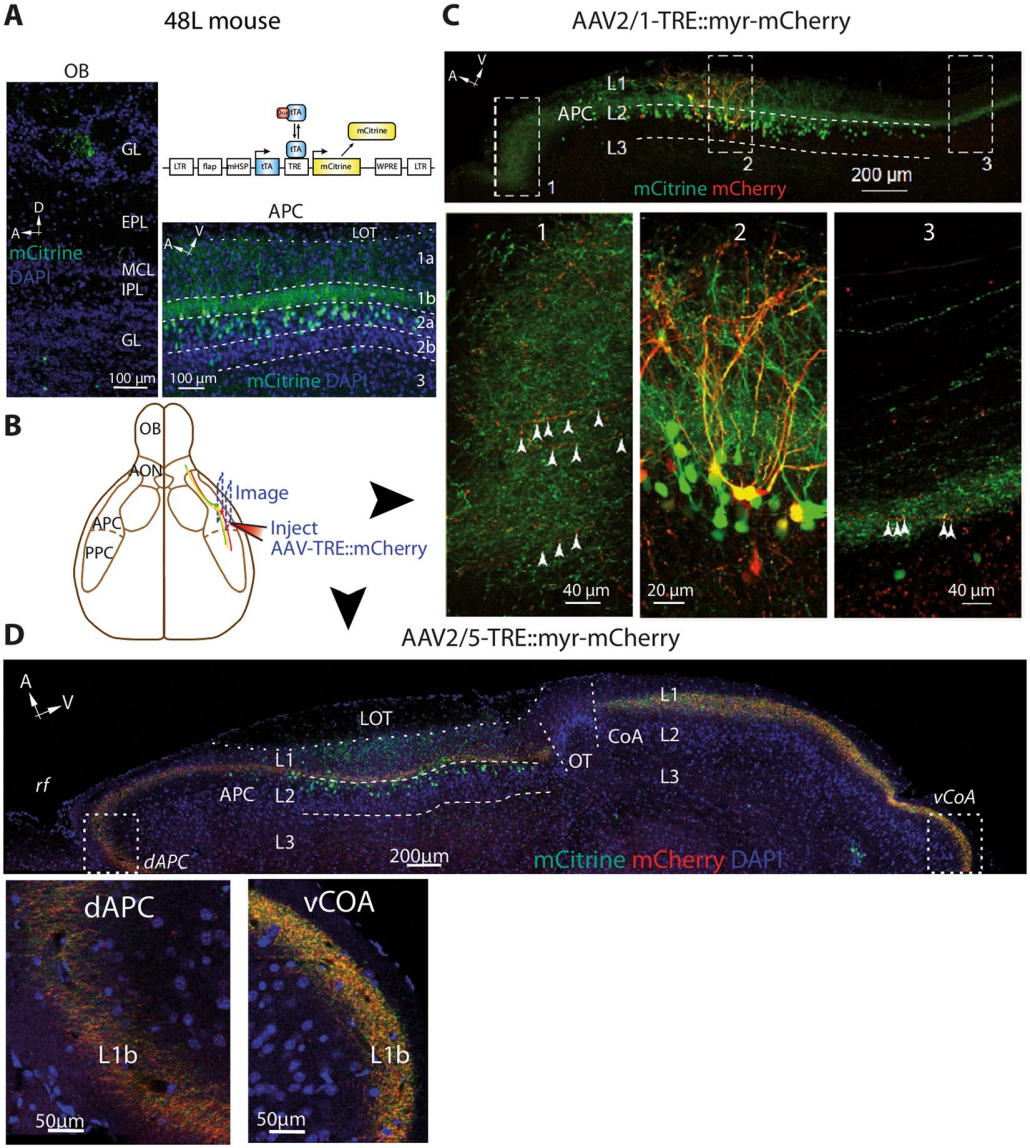
SL cells project widely within the olfactory system as revealed by a transgenic mouse line, 48L. (**A**) tTA constitutionally binds to TRE and drives mCitrine expression in a subset of SL cells (dox off system; *up right*). mCitrine^+^ cells were concentrated in layer 2a of the APC (*bottom right*). In the OB (*left*), some cells were observed in the glomerular and granule cell layers and axons were rarely observed. GL: glomerular layer, EPL: external plexiform layer, MCL: mitral cell layer, IPL: internal plexiform layer, GCL: granule cell layer. (**B**) AAVs expressing mCherry under the control of TRE and tTA were injected in the APC to yield sparse dual-labeling and identification of dual-labeled axons away from the injection site. (**C**) AAV2/1-TRE::myr-mCherry injection in the APC led to sparse dual-labeling of mCitrine^+^ cells (box 2). Dual-labeled axons were found several hundred µm away from the injection site in the same sagittal plane, in the dorsal (box 1) and ventral APC (box 2). n = 2 mice. (**D**) AAV2/5-TRE::myr-mCherry injection in the APC labeled axons several hundred µm away from the injection site, in a parallel plane. Dual-labeled axons were found in the dorsal APC and in the CoA. OT: Olfactory Tubercle. For B and C, red: mCherry. Green: mCitrine. Blue: DAPI. n = 3 mice.

**Figure 5:**
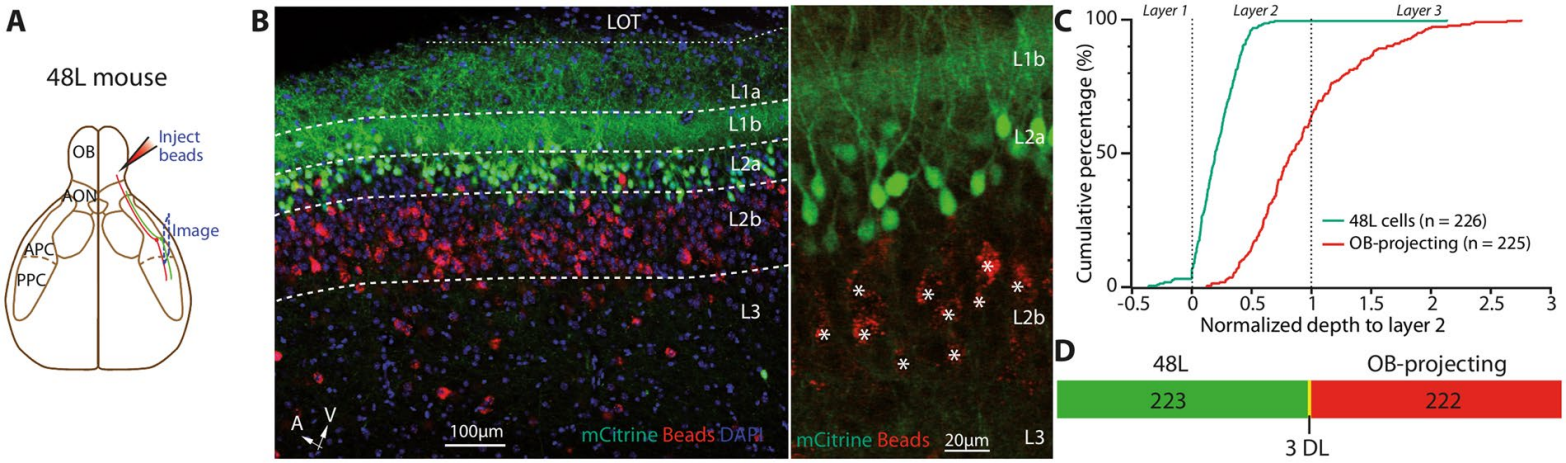
Genetically labeled SL cells do not send feedback projection to the OB. (**A**) Schematic representation of the injection strategy. Red Retrobeads were injected into the OB of 48L mouse. Images were taken from the APC. (**B**) Red bead injections in the OB of 48L mouse failed to labeled mCitrine^+^ cells, indicating that the genetically tagged subset of SL cells is not projecting back to the OB (3 dually-labeled cells for 225 Retrobeads + cells and 226 mCitrine + cells, 3 sections, 2 mice). Middle and right panels are extracted from different experiments. Stars in right panels indicate Retrobeads^+^ OB-projecting cells. Red: Retrobeads. Green: mCitrine. Blue: DAPI. (**C**) Cumulative distribution of 48L-labeled cells and Retrobead-labeled cells in the APC (n = 225 Retrobeads + cells, 226 mCitrine + cells, 3 sections, 2 mice). (**D**) Venn diagram showing the near-zero overlap of 48L cells and OB-projecting neurons.

We showed that a small fraction of layer 2a neurons in the APC can project to the OB or PPC (Figs 1C–F and 2C–E). Neurons in Layer 2a are mainly composed of SL cells, believed to be specialized in providing feedforward excitation to pyramidal cells of layer 2b and layer 3^15–17^. However, one study involving single neuron tracing reported that layer 2a cells can extend axons to multiple olfactory regions^21^ and a recent work combining genetics and tracing techniques showed that layer 2a cells project to distinct brain regions^22^. Therefore, we took advantage of a mouse line in which SL cells specifically express the reporter protein mCitrine and tetracycline activator (tTA) (48L mouse line, mCitrine expressed in 46 ± 2% of L2a Nissl-labeled cells, from ref. 17)^17, 37^ to investigate the projection pattern of SL cells (Fig. 4A). Labeled mCitrine^+^ axons were found throughout upstream and downstream olfactory cortical areas including the AON, PPC, and CoA, while very few axons were labeled in the OB (Fig. 4A **)**, as previously reported for generic layer 2a neurons that were not genetically identified^21, 22^. To ensure labeled axons were bona-fide projections from SL cells located within the APC, we injected AAVs to express myr-mCherry under TRE promoter, which is activated by tTA expressed in SL cells. A survey of different serotypes of AAVs by injection into the APC showed that AAV2/5 has the highest, while AAV2/8 has the lowest, labeling efficiency for SL cells (Supplementary Fig. S6A). Notably, diluted AAV2/1 injections (AAV2/1-TRE::myr-mCherry; Fig. 4B) in the APC led to sparse dual labeling of a SL cell subpopulation. Individual double-labeled axons projected at least 1 mm away from APC in both the anterior and posterior directions (Fig. 4C). Furthermore, larger volume injections of AAV2/5 in the same location labeled SL-mCitrine^+^ axons in various olfactory cortical regions including the AON, APC, and CoA (Fig. 4D), but not in the OB. To directly visualize whether projections of SL cells spare the OB, we injected red Retrobeads in the OB of 48L animals (Fig. 5A). There was a near absence of co-labeling between the genetic reporter of SL cells mCitrine and the injected Retrobeads (3 dual-labeled cells out of 226 48L- and 225 bead-labeled cells; Fig. 5B–D **)**. On the other hand, bead injection into the PPC of 48L mouse revealed some dual-labeled cells in L2a (Supplementary Fig. S6B). Taken together, our data show that SL cell population extends long-range projections to multiple olfactory cortical areas (such as the AON and the PPC). By contrast, SL cells appear to not send feedback projections to the OB.

## Discussion

In this study, we injected retrograde tracers and AAVs in the OB and PPC of wild-type and transgenic mice to examine the distribution as well as the projections of principal neurons from the APC. We characterized the laminar distribution of projecting populations and identified a substantial fraction of neurons dually projecting to the OB and the PPC. In addition, we showed that genetically labeled SL cells project to numerous brain regions, but not back to the OB. These findings bring new knowledge about how the APC broadcasts olfactory information to the brain, and future studies using optophysiological methods such as ChR2-assisted circuit mapping will enhance our understanding of whether the circuits highlighted here exhibit particular rules of connectivity.

We found that OB-projecting neurons were mostly present in layers 2 and 3 of the APC, while PPC-projecting neurons were found mainly in layer 2 of the APC and in the EndoP. Within layer 2, both OB- and PPC-projecting populations were largely skewed toward layer 2b. Recent work from Diodato and colleagues^22^ found a similar distribution of OB-projecting neurons, and also reported a layer 2b-biaised distribution of APC neurons projecting to the medial prefrontal cortex. When the proportion of projecting cells were corrected for variations in cell densities among layers, layer 2b cells were still the prominent source of projections to the OB (Fig. 1E). This suggests an internal bias toward layer 2b cells for APC feedback projections to the OB. Layer 2 of the APC is composed of superficial layer 2a, mainly populated by SL cells, and deep layer 2b, mainly containing SP cells – although a continuum exists between the two cell populations^15^. SL cells receive strong bottom-up inputs from the OB and form little or no recurrent connections with local excitatory neurons^15–17^. In contrast, SP cells receive stronger inputs from recurrent axons and project outside the APC^15, 16, 38^. Our data and previous work show that the main projection channel of the APC indeed originates from layer 2b, presumably from SP cells. However, in this study, we also genetically labeled a significant proportion of projecting layer 2a cells that were shown previously to be SL cells based on morphological and electrophysiological characterization^17^. Genetic labeling revealed that SL cells do send axonal projections to multiple olfactory regions, which corroborates with a single-cell tracing study examining SL cell projections outside the APC^21^. In an earlier work using retrograde tracer injections in the CoA or LEnt, Diodato and coworkers^22^ identified layer 2a as the main APC output channels to these brain regions. Therefore, it appears that SL and SP cells of layer 2 constitute two parallel output channels of the APC. It is possible that SL cells send odor information that received little local processing in APC whereas SP cells send more processed signals owing to the extensive recurrent connections.

Strikingly, while the genetic labeling of SL cells revealed projections to a variety of brain regions, it failed to reveal significant feedback projections to the OB (Figs 4 and 5). We did observe very few axons in the OB, but these projections were very sparse and likely originates locally from labeled cells in the OB (juxtaglomerular cells in the glomerular layer or cells in the granule cell layer^17^). However, since the 48L mouse line labels approximately half of SL cells in L2a^17^, we cannot exclude the possibility that some unlabeled SL cells do project to the OB because ~20% of OB-projecting cells reside in L2a (Fig. 1E). In addition, injection of a TRE-dependent virus into the APC to express mCherry ensures the neurons and axons labeled in the AON, APC and CoA (Fig. 4) are genuinely originating from SL cells residing in APC. Here, we propose a circuit model where SL and SP cell projections are organized differently depending on whether these are feedback or feedforward motifs. Since SL cells receive stronger inputs from the OB and are basically not recurrently connected^39^, they are the first processing station in the APC. Information is fed forward from SL cells to higher olfactory regions as well as to SP cells within the APC. For feedback information, however, additional processing seems to be required: SL to SP and SP to SP connections will dictate the kind of information sent back to the OB. Since SL cells do not project back to the OB but instead rely on SP cells to relay feedback information, this can form a hierarchical processing circuit. On the other hand, feedforward/recurrent processing can occur in a parallel fashion for SL and SP outputs (Figs 2 and 4). Although our methods reveal important projection differences between SL and SP cells, they do not provide any information about synaptic connectivity. Further quantitative anatomy and optophysiological mapping of connectivity will provide insights into how these cell types are connected with upstream and downstream regions.

Within the APC, OB- and PPC-projecting neurons were found mainly in layer 2. Dual-labeling experiments showed that a sizeable fraction of OB- or PPC-projecting neurons actually project to both areas. Our results of empirical fractions of overlap (11.6 to 18.2%, Fig. 3) likely represent an underestimate of the actual overlap. This is because although our bead injections generally resulted in high labeling efficiency (colabeling of ~90% when injected into the same site in OB; Fig. S4), the fragmented spatial organization of centrifugal fibers rendered it difficult to label a large fraction of axons and neurons. Therefore, it appears that information emerging from these L2b SP cells, and thus similar odor representation, is simultaneously sent back to the OB and forward to the PPC. In contrast, using a similar dual-tracing technique, Chen and colleagues^34^ identified distinct OFC-projecting neuronal populations in the APC, although spatially intermingled. Genetic analysis of different projecting populations^22^ further shows that neuronal identity (marker expression) is more important than neuronal location in determining which brain regions these axons will target. Additional connectivity studies, which might benefit from tissue clearing techniques, are necessary to gain insight on whether APC outputs are predominantly multiplexed or rather parallelized into distinct channels. We believe that a better understanding on odor coding in the brain requires elucidation of the output organization of the APC. It is likely that the formation of odor percepts involves wide recruitment of multiple brain areas and intricate feedback and feedforward circuits.

## Methods

### Animals

C57Bl/6J, CaMK2a-Cre^40^ and 48L mice (labeling SL cells) were used in this study. All experiments were performed in accordance with the guidelines set by the National Institutes of Health and approved by the Institutional Animal Care and Use Committee at Harvard University.

### Retrograde labeling

In this study, non-viral tracers (green and red fluorophore-coated latex Retrobeads; Lumafluor), and viral tracers were used to retrogradely label APC projecting neurons.

For OB retrograde labeling (Fig. 1 and Supplementary Fig. S1), the viruses used were: AAV2/1-CAG-hChR2(H134R)-mCherry, AAV2/8-CAG-ChR2-GFP, AAV2/9-CAG-ChR2-Venus, AAV2/8-CAG-Flex-EYFP, and AAV2/9-Flex-ChR2-eYFP. All viruses were purchased from the Penn Vector Core. Cre-dependent viruses were used in CaMK2a-Cre mice while and non-Cre dependent viruses were injected in C57BL/6J mice. For APC injections in 48L mice (Fig. 4 and Supplementary Fig. S4), we used AAV-TRE::myr-mCherry with capside serotype 2/1 or 2/5.

Briefly, adult male mice (1 to 4 months old) were deeply anesthetized with an intraperitoneal injection of ketamine/xylazine mixture (100 mg/kg and 10 mg/kg, respectively) and placed in a stereotaxic apparatus. A small craniotomy was performed above the injection site and labeling solution was injected into the OB, APC or PPC using a glass pipette (Drummond Wiretrol 5-000-1001). The following are the coordinates for injections. OB (from junction of inferior cerebral vein and superior sagittal sinus): AP 1.2 mm, ML 1.1 mm, DV –1 mm; volume injected: 50 nL Retrobeads or 300 nL virus, or otherwise stated in the article. APC (from bregma): ML 2.6–2.8 mm, AP 1.3–1.5 mm, DV –3.5 mm, 50 nL of Retrobeads or 100–300 nL of viral solution. PPC (from bregma): ML 3.4–3.9 mm, AP 0.3–0.7 mm, DV -4.4–4.9 mm from brain surface, 50 nL of Retrobeads.

### Histology and cell counting

1 to 4 days after Retrobead injections or two weeks after viral injections, mice were perfused intracardially with 4% v/v paraformaldehyde and brains were post-fixed in the same fixative overnight. 100 µm-thick brain sections were cut with a vibratome (Leica VT1000 S), rinsed in PBS, counterstained with the nuclear dye 4,6-diamidino-2-phenylindole (DAPI) and mounted on slides. Z-stack confocal images were taken with a Zeiss LSM 780 or 880 confocal microscope. The size of pinhole was adjusted to yield optical slice depth of approximately 10 µm to ensure that we do capture single neurons by cross-examining images in a thicker Z-stack. Counting was performed over the full D-V length of the APC, excluding the region below the rhinal fissure and where layer 2a and 2b could not be clearly identified. 2 to 3 sections per animal were taken (sagittal sections, 200–400 μm apart) and fluorescent cells were manually counted with the Fiji plugin “Cell Counter” by Kurt de Vos (University of Sheffield) on single-plane images.

For the quantification of bar graphs in Figs 1 and 2, APC was separated into 4 sublayers manually: layer 1, 2a, 2b and 3/endoP. Since there was not a clear boundary between layer 3 and endoP, we analyzed them as one. For each sublayer, the percentage of labeled cells was defined as the number of labeled cells divided by the number of DAPI + cells in that area. This normalization accounts for variations in cell density in sublayers. Since both labeled cells and DAPI + cells were counted in the same area, this normalization does not depend on area size. Next, the total percentage was calculated by adding up all 4 sublayer percentage values. The fraction of labeled cells reported for each sublayer is the sublayer percentage divided by the total percentage. The sum total of all 4 fractions is 1. This normalization accounts for variations in injection/labeling efficiency.

For cumulative plots, the depth of the cells in layer 2 was determined by measuring the distance of a cell to the layer 1/layer 2 border (defined as zero) and reported to the depth of layer 2 in that region (defined as one) using custom MATLAB scripts. DAPI + cells were quantified on the binarized image in the middle of the Z-stack. The mean of DAPI + cells across experiments was used for normalization in bar graphs.

### Statistics

All results are given as mean ± standard error of the mean (SEM). All statistical tests were performed using commercial analysis software (Graphpad Prism) or custom script in MATLAB with a 5% significance level (see Supplementary Table online).

## Electronic supplementary material

Supplementary information
