# Supplementary material for: Distinct projection patterns of different classes of layer 2 principal neurons in the olfactory cortex
Source: Sci Rep. 2017 Aug 15;7:8282. doi: 10.1038/s41598-017-08331-0 (PMC5558010; doi:10.1038/s41598-017-08331-0)
Supplement: Supplementary file 1 — Supplementary information [file 41598_2017_8331_MOESM1_ESM.pdf]

Supplementary Material

Distinct projection patterns of different classes of layer 2 principal neurons in the olfactory cortex

Camille Mazo[1,2], Julien Grimaud[1,2], Yasuyuki Shima[3], Venkatesh N. Murthy[1*], C. Geoffrey Lau[1,4*].

1. Department of Molecular and Cellular Biology and Center for Brain Science, Harvard University, Cambridge, MA, USA.

2. Ecole Normale Supérieure de Cachan, Université Paris-Saclay, F-94235, Cachan, France

3. Department of Biology and National Center for Behavioral Genomics, Brandeis University, Waltham, MA 02454, USA.

4. Department of Biomedical Sciences and Centre for Biosystems, Neuroscience, and Nanotechnology, City University of Hong Kong, 83 Tat Chee Avenue, Kowloon, Hong Kong.

* Corresponding authors. V.N.M., vnmurthy@fas.harvard.edu. C.G.L., geoff.lau@cityu.edu.hk.

6 Supplementary Figures and Supplementary Figures Legends

1 Supplementary Table

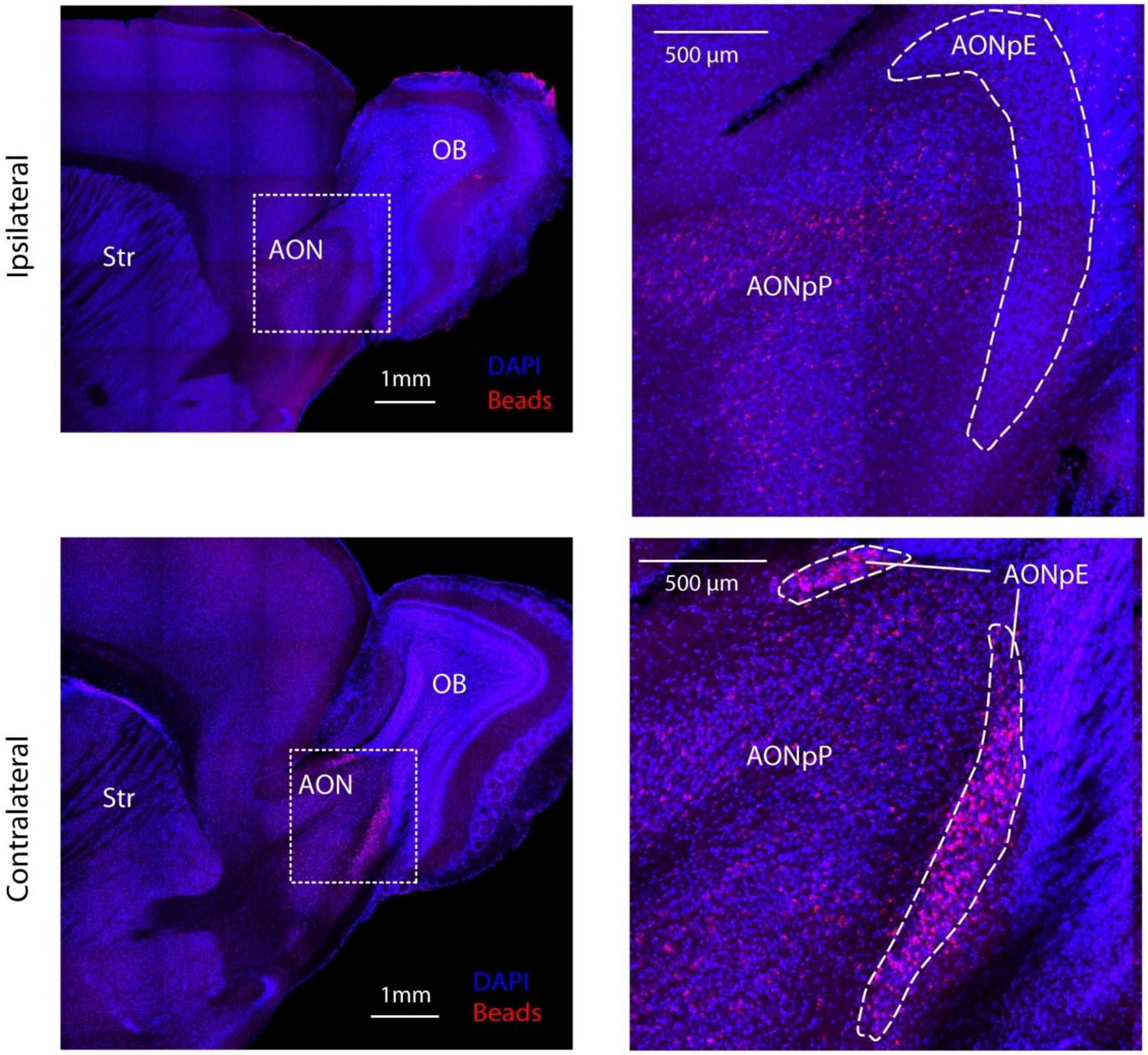

Figure S1. OB bead injections labeled AON pars externa contralateraly, but not ipsilateraly.

Low (left) and high (right) magnification of the AON. *Up*, Ipsilaterally, bead$^+$ cells were present in the AON pars principalis (AONpP), but absent in the AON pars externa (AONpE). *Bottom*, In contrast, bead$^+$ cells were found across both contralateral AON subdivisions, and denser labeling was observed in the AON pars externa. Red: beads. Blue: DAPI. This confirms the validity of our retrograde labeling approach. Str, striatum; AOE, AON pars externa.

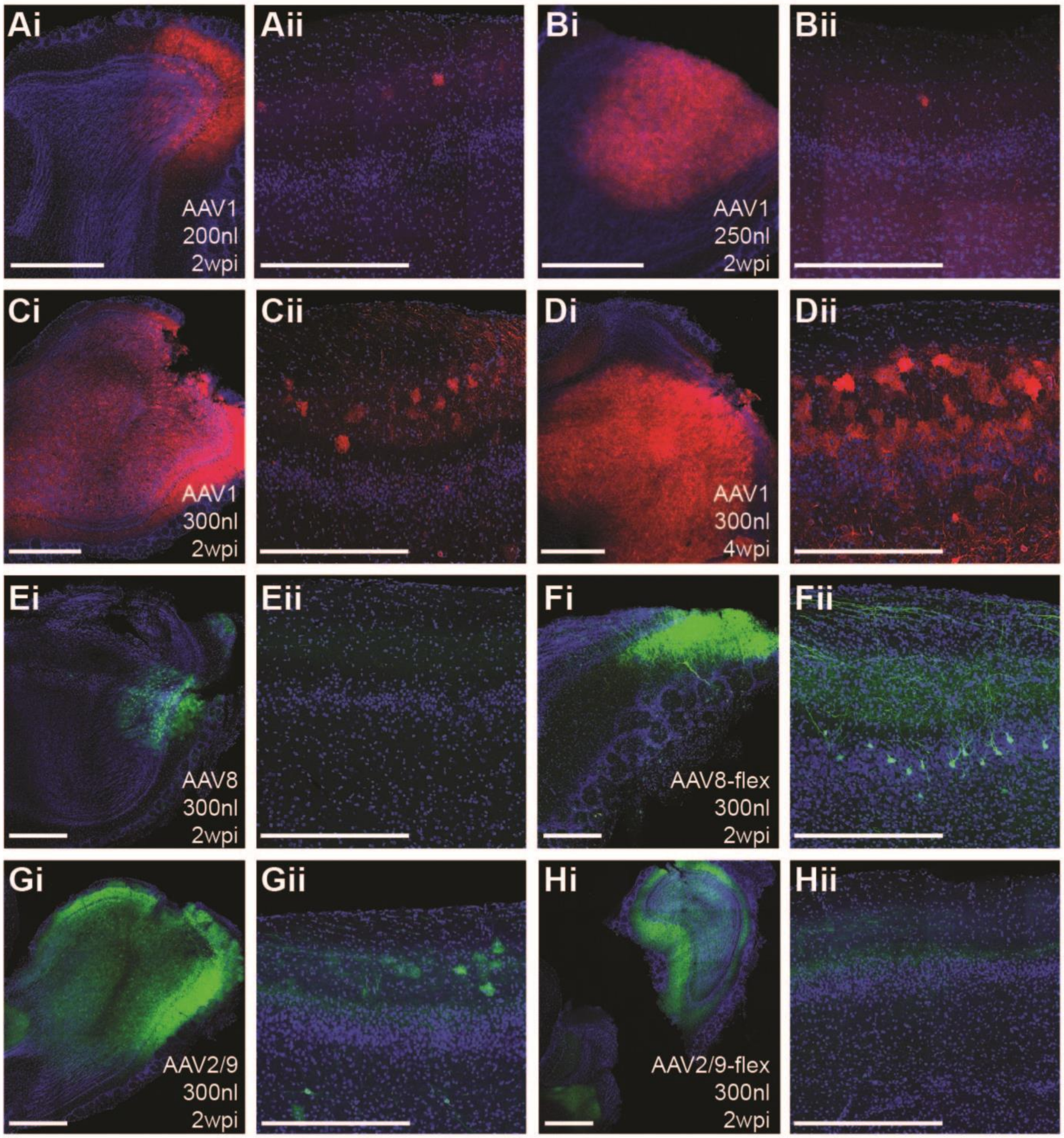

Figure S2. Differential ability of AAV serotypes to drive retrograde labeling of APC neurons.

A-H: Different AAV serotypes were injected in the OB and retrograde labeling of APC neurons was observed. 2 mice were used per AAV serotype. For each trial, (i) is a sagittal section of the OB (on the top: dorsal, on the right: anterior), and (ii) is a sagittal section of the APC (on the top: ventral, on the right: posterior). Scale bar: 500µm. Blue: DAPI. Red: mCherry. Green: Venus for (G), EGFP for (H), EYFP otherwise. wpi: weeks postinjection (number of weeks between injection and fixation). The volume written on each picture is the injected volume of AAV. AAV2/8-Flex-eYFP was the only virus to yield significant retrograde labeling.

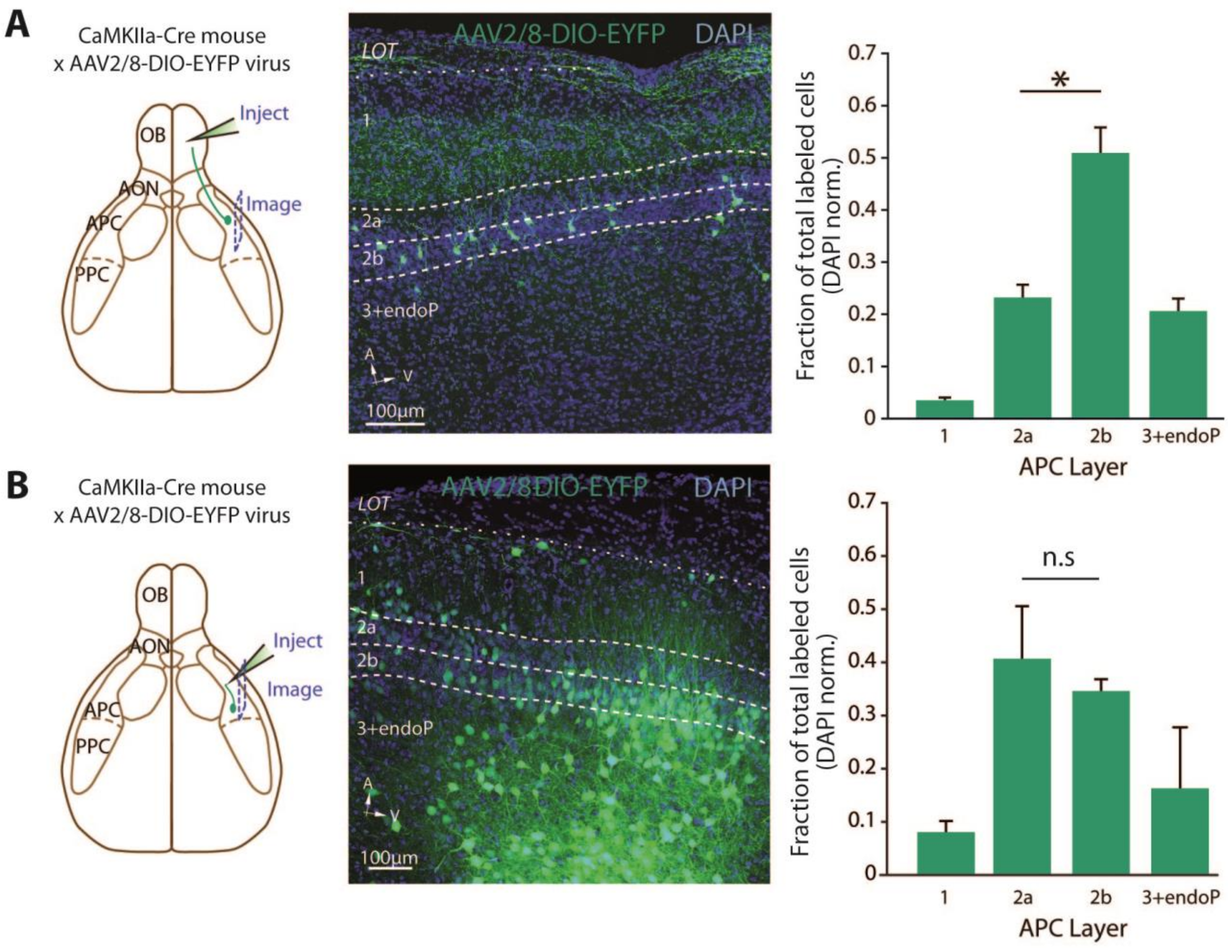

Figure S3. AAV-mediated retrograde labeling confirms that OB-projecting cells predominantly originate from layer 2b.

A. *Left*, Cre-dependent AAVs were injected into the OB of CaMKII-Cre mouse and images were taken from the APC (*middle*). Layer 2b cells were more densely labeled retrogradely in APC by injection of AAV2/8-DIO-EYFP into the OB (*right)* (total 213 EYFP+ cells, 10,664 DAPI cells, n = 9 sections, 3 mice). *, $p < 0.05$. B. Cre-dependent AAV injection and subsequent imaging in the APC of CaMKII-Cre mouse (*left* and *middle*). Layer 2a and 2b cells were equally labeled using direct AAV2/8-DIO-EYFP injection into the APC (*right)* (total 343 cells EYFP+ cells, n = 3 sections, 1 mouse). n.s, not significant. Green: AAV2/8-DIO-EYFP; Blue: DAPI; LOT: lateral olfactory tract; endoP: endopiriform nucleus.

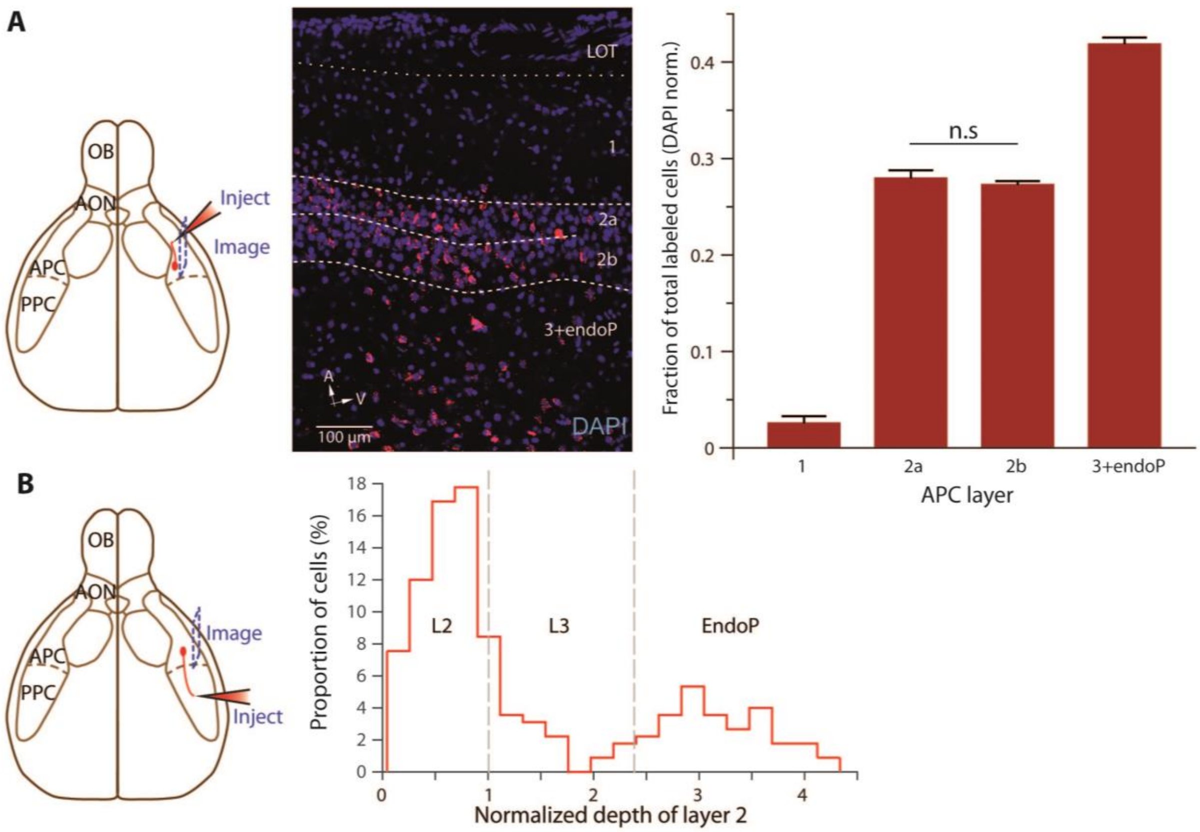

Figure S4. Bead injections show bimodal distribution of PPC-projecting cells and demonstrates the unbiased labeling of SL and SP cells in the APC.

A. *Left*, Red bead injection and subsequent imaging in the APC. *Middle and right*, Retrogradely labeled cells were found equally in layer 2a and 2b (total 698 beads+ cells, 1,196 DAPI+ cells, n = 4 sections, 2 mice). n.s, not significant. B. *Left*, Red beads were injected in the PPC and images were taken from the APC. *Right*, Distribution of the PPC-projecting cells (not normalized for DAPI distribution) across APC layers showing a substantial proportion of retrogradely-labeled cells in the EndoP (total 581 beads+ cells, n = 5 sections, 4 mice).

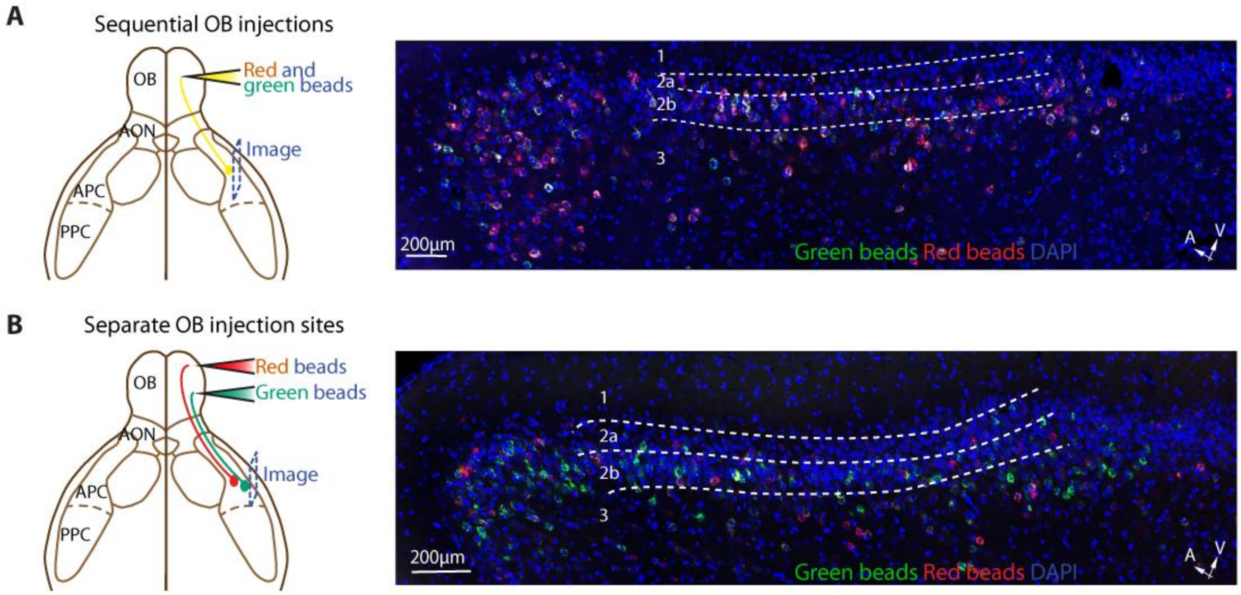

Figure S5. Single bead injections allow robust labeling of APC cells but topography of feedback projections restricts the labeled population.

A. Equal amounts of green and red beads were sequentially injected in the OB, leading to substantial dual-labeling in the APC (90.5 - 92.7%, total 205 – 210 cells, n = 2 sections, 2 mice). B. Green and red beads were injected ~500 µm apart in the OB, leading to seldom dual-labeling in the APC (21.1 – 34.3%, n = 67 – 109 cells, n = 2 sections, 2 mice). Red: red beads. Green: green beads. Blue: DAPI.

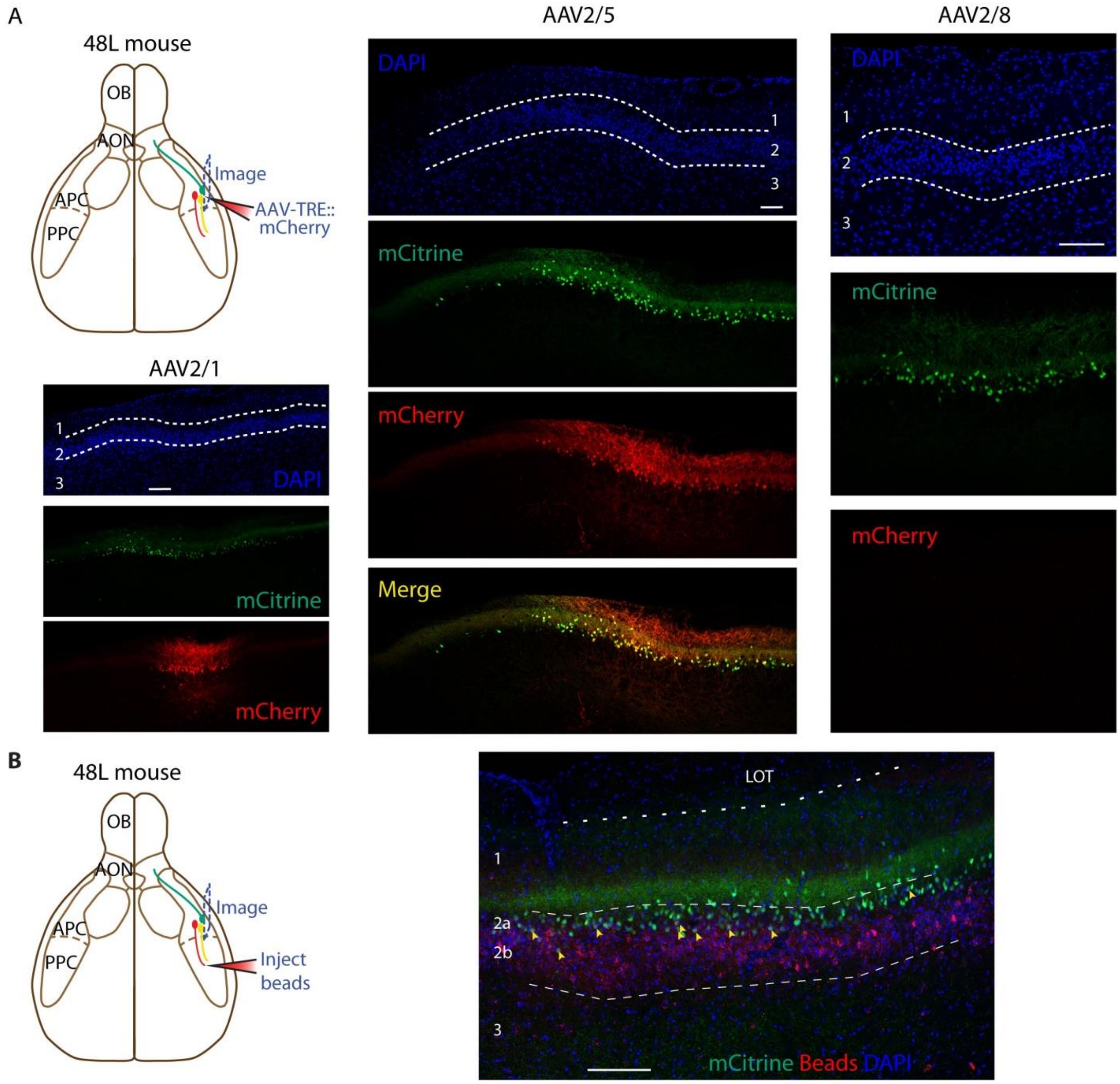

Figure S6. Genetic labeling of a subset of SL cells allows further investigations of an APC output channel.

A. Differential ability of AAV vectors to infect APC neurons depending on the capsid serotype. AAV2.5 drives the best expression while AAV2.8 failed to labeled neurons. n = 2 mice per condition. B. mCitrine^{+} and beads^{+} PPC-projecting neuron distributions are mostly spatially segregated but a sizeable portion of dual-labeled neurons could be found. Green: mCitrine. Red: tracer. Blue: DAPI. n = 3 sections, 2 mice. Scale bars are 200 µm.

Experiment *(mouse line Type of experiment)*	Corresponding Figure	retrogradely-labeled cells #	DAPI+ cells #	48L cells #	# of sections	# of mice	Statistic test
**WT Beads injections in OB**	1E	546	4474	*NA*	13	11	Friedman Dunn's multiple comparisons
	1F	1454	658	*NA*	27	12	KS
	S1					12	*NA*
**WT Beads injections in PPC**	2D, S4B	581	2529	*NA*	5	4	Friedman Dunn's multiple comparisons
**CaMKII-Cre Other AAV injections in OB**	S2	*NA*	*NA*	*NA*	*NA*	2/virus	*NA*
**CaMKII-Cre AAV8 injections in OB**	S3A	213	10 664	*NA*	9	3	Friedman Dunn's multiple comparisons
**CaMKII-Cre AAV8 injections in APC**	S3B	343	912	*NA*	3	1	Wilcoxon ranksum matched-pairs
**WT Beads injections in OB and PPC**	2E, 3C	499	658	*NA*	12	5	Friedman Dunn's multiple comparisons KS
**WT Beads injections in APC**	S4A	698	1916	*NA*	4	2	Wilcoxon ranksum matched-pairs
**WT OB sequential injections**	S5A	415	*NA*	*NA*	2	2	*NA*
**WT OB injections 500µm appart**	S5B	176	*NA*	*NA*	2	2	*NA*
**48L AAV2/1 injections in APC**	4C	*NA*	*NA*	*NA*	*NA*	2	*NA*
**48L AAV2/5 injections in APC**	4D	*NA*	*NA*	*NA*	*NA*	3	*NA*
**48L Other AAV injections in APC**	S6A	*NA*	*NA*	*NA*	*NA*	2/virus	*NA*
**48L Beads injections in OB**	5C	225	*NA*	226	3	2	KS
**48L Beads injections in PPC**	S6B	*NA*	*NA*	*NA*	3	2	*NA*

Supplementary Table: Descriptive statistics. NA: Not applicable. KS: Kolmogorov-Smirnov test
